# Evaluation of Prognostic Implication of Serum Mixed Lineage Kinase Domain‐Like Protein in Acute Primary Supratentorial Intracerebral Hemorrhage: A Multicenter Prospective Cohort Study

**DOI:** 10.1002/brb3.70424

**Published:** 2025-03-13

**Authors:** Yijun Ma, Jun Wang, Chao Tang, Jin Liu, Xiaoyu Wu, Xiaoqiao Dong, Quan Du, Wei Li, Xuan Lv, Suijun Zhu

**Affiliations:** ^1^ Department of Neurosurgery First People's Hospital of Linping District Hangzhou China; ^2^ Department of Neurosurgery, Linping Campus the Second Affiliated Hospital of Zhejiang University School of Medicine Hangzhou China; ^3^ Department of Neurosurgery The Sixth Affiliated Hospital of Wenzhou Medical University Lishui China; ^4^ Department of Neurosurgery The Affiliated Hangzhou First People's Hospital, Westlake University School of Medicine Hangzhou China

**Keywords:** early neurological deterioration, intracerebral hemorrhage, mixed lineage kinase domain‐like protein, outcome, prognosis, severity

## Abstract

**Objective:**

Mixed lineage kinase domain‐like protein (MLKL) is a key component of necroptosis. Here, serum MLKL levels were measured with the intent to assess its prognostic significance in acute intracerebral hemorrhage (ICH).

**Methods:**

A collective of 161 patients with acute primary supratentorial ICH and 73 controls were enlisted in this multicenter prospective cohort study. Serum MLKL levels were measured at admission in all patients, at study entry in all controls, and on post‐ICH days 1, 3, 5, 7, 10, and 15 in 73 of all patients. Multivariate analyses were adopted to assess relationships between serum MLKL levels, severity, early neurological deterioration (END), poststroke 6‐month modified Rankin Scale (mRS) scores, and poor prognosis (mRS scores of 3–6).

**Results:**

Patients, relative to controls, had significantly promoted serum MLKL levels from admission until Day 15, with the peaking value at Day 3 (*p* < 0.001). Admission serum MLKL levels were independently correlated with National Institutes of Health Stroke Scale (NIHSS) scores (beta, 0.133; 95% confidence interval (CI), 0.088–0.178; *p* = 0.011), hematoma volume (beta, 0.051; 95%CI, 0.037–0.064; *p* = 0.001), and 6‐month mRS scores (beta, 0.707; 95%CI, 0.487–0.927; *p* = 0.023), as well as independently predicted END (odds ratio, 1.902; 95%CI, 1.229–2.945; *p* = 0.014) and poor prognosis (odds ratio, 2.286; 95%CI, 1.324–3.946; *p* = 0.038). Admission serum MLKL levels were linearly connected to risks of poor prognosis (*p* > 0.05) and END (*p* > 0.05), had no interactions with age, gender, hypertension, and so forth (all *p* > 0.05), and possessed similar areas under the receiver operating characteristic curve to NIHSS scores and hematoma volume (all *p* > 0.05). The models integrating serum MLKL levels, NIHSS scores, and hematoma volume were graphically represented by nomogram and predicted END and poor prognosis with a good consistency under the calibration curve.

**Conclusions:**

Serum MLKL levels are markedly increased shortly following ICH, and may accurately mirror disease severity, and efficaciously anticipate END and six‐month bad prognosis of patients, strengthening serum MLKL as a prognostic biomarker of good prospect in ICH.

## Graphical abstract

Serum MLKL levels are immediately elevated until Day 15 after ICH. Serum MLKL levels are firmly linked to severity, early neurological deterioration and a poor outcome at the 6‐month mark following ICH. In summary, serum MLKL appears as a prognostic indicator of ICH.

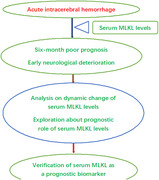

**Abbreviations**
ICH: intracerebral hemorrhage
NIHSS: National Institutes of Health Stroke Scale
ROC: receiver operating characteristic curve
CT: computed tomography
END: early neurologic deterioration
AUC: area under curve
OR: odds ratio
95% CI: 95% confidence interval
MLKL: mixed lineage kinase domain‐like protein
TNF: tumor necrosis factor
RIPK: receptor‐interacting protein kinase.

## 1 Introduction

Primary intracerebral hemorrhage (ICH), a lethal form of the frequently encountered disease, ranks second in incidence while taking possession of the highest mortality among all strokes (Romero and Rojas‐Serrano 2023). Aside from supportive therapeutic modalities, no specific remedies have been exploited for relieving neurological sequelae of patients (Seiffge and Anderson 2023). In view of pathophysiological mechanisms, the occurrence of primary ICH is principally attributed to hypertensive arteriopathy and cerebral amyloid angiopathy (Y. Zhu et al. 2023; Roh et al. 2018). Vascular rupture, as a result of vasculopathy in conjunction with blood pressure fluctuation, facilitates the intraparenchymal accumulation of hematoma, thereby elevating intracranial pressure, disrupting the blood‐brain barrier, activating the release of inflammatory mediators, aggravating brain edema, inducing cell death, and finally leading to neurological impairments and even patient death (Sutherland and Auer 2006). Clinically, early neurological deterioration (END) is recognizable as a very common adverse affair subsequent to ICH and its emergence tremendously enhances the likelihood of poor prognosis in ICH (W. Zhu et al. 2024). Hematoma volume and the National Institute of Health stroke scale (NIHSS) harbor extensive applicative values in the prognostic assessment of ICH (Carhuapoma et al. 2024). Also, some routine blood markers, such as blood leucocyte counts, blood C‐reactive protein levels, and blood glucose levels, have been extremely studied with respect to their prognostic ability in ICH (J. Liu, Luo, et al. 2024; Y. Liu, Qiu, et al. 2024; Yang et al. 2024). However, the final results are unsatisfactory because these biomarkers do not hold more valuable prognostic capability (de Liyis et al. 2024; Sasongko et al. 2025). For the sake of picking better prognostic indices, the exploration of blood biomarkers as the prognostic indicators of ICH has been broadly emphasized during recent decades (Hu et al. 2023; Yan et al. 2022; Wang et al. 2022).

Necroptosis is classified as a kind of programmed necrosis, which is manifested as resultant cell leakage following cell swelling and subsequent plasma membrane disruption, thereby provoking the immune system and strongly incurring proinflammatory effects (Shi et al. 2024). Necroptosis is involved in secondary brain injury subsequent to traumatic brain injury, ischemic stroke, ICH, and subarachnoid hemorrhage (L. Zhang et al. 2024). Mixed lineage kinase domain‐like protein (MLKL), along with receptor‐interacting protein kinase (RIPK)‐1 and ‐3, are the three crucial components of necroptosis (Nakano 2024). In experimental ICH, MLKL was strongly expressed by astrocytes and neurons (Yuan et al. 2023; Huang et al. 2022; Lule et al. 2021). Moreover, neuronal death was increased, blood‐brain barrier permeability was improved, and neurological deficits were mitigated in MLKL‐deficient mice (Lule et al. 2021). Also, via inhibiting MLKL, neuroinflammation was decreased, brain edema was attenuated, brain infarctional area was diminished and subsequently, neurological functions were recovered in rats subjected to acute ischemic stroke (Zhou et al. 2017). These converging data support MLKL as an endogenous deleterious factor for maximizing secondary brain injury, alluding to the point that MLKL may be a brain injury biomarker. Here, serum MLKL levels were measured in an attempt to investigate the prognostic of serum MLKL in ICH.

## 2 Methods and Materials

### 2.1 Study Design and Ethical Approval

In this multicenter observational analytic study between February 2022 and May 2023, ICH patients were consecutively recruited from the following three hospitals, namely, the Affiliated Hangzhou First People's Hospital, Westlake University School of Medicine (Hangzhou, China), the First People's Hospital of Linping District (Hangzhou, China), and the Sixth Affiliated Hospital of Wenzhou Medical University (Lishui, China). Specifically, this study encompassed two sub‐studies. One was a cross‐sectional study, which was performed to investigate longitudinal change of post‐ICH serum MLKL levels. In this part, serum MLKL levels were quantified at study entry in all controls, as well as at admission and at days 1, 3, 5, 7, 10, and 15 after ICH in a fraction of patients who were permitted blood‐drawings at multiple time‐points. The other was a prospective cohort study, which was done to determine the predictive role of serum MLKL on post‐ICH END and six‐month poor prognosis. In this part, serum MLKL levels were detected at admission in all patients. The current study was designed in compliance with the tenets of the Declaration of Helsinki and local legislation for human research. The approval to study protocol was granted from the ethical committees at the preceding three hospitals (No. 058‐01, LPH2018012, 2020‐001). Written informed consents were gained from patients’ proxies or controls themselves.

### 2.2 Participant Enrollments

**FIGURE 1:**
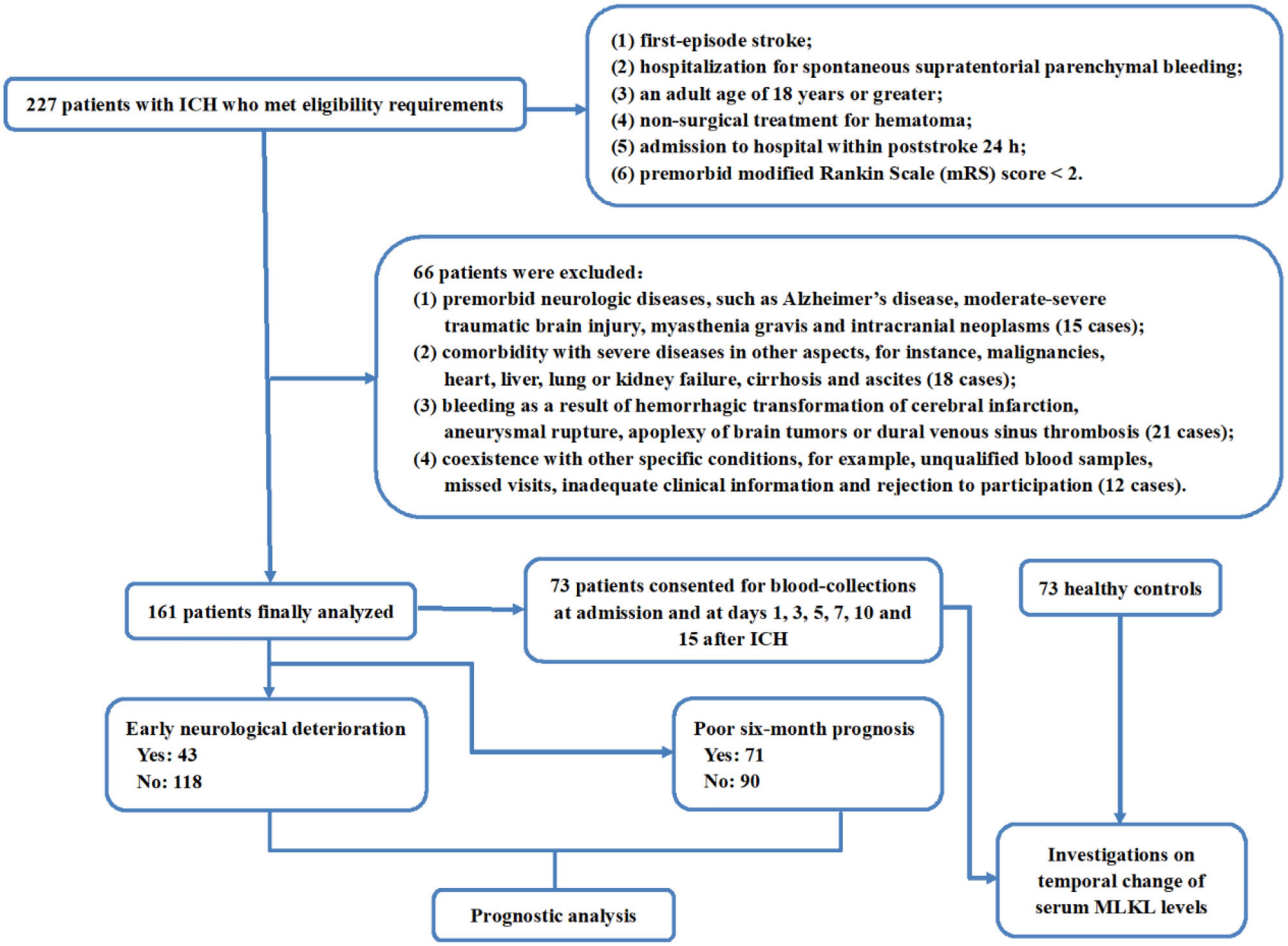
Study‐design diagram. A cohort of 227 patients were initial assessed, and 66 patients were excluded, leading to a final group of 161 patients. From this pool, 73 patients allowed to supply blood‐samplings at multiple time points. A total of 73 healthy controls were selected. Here, dynamic alteration of serum lineage kinase domain‐like protein levels after intracerebral hemorrhage was determined and prognostic analysis was done. ICH, intracerebral hemorrhage; MLKL, mixed lineage kinase domain‐like protein; mRS, modified Rankin Scale.

ICH was confirmed via the head computed tomography (CT) scan. ICH patients were firstly demanded to experience a first‐episode stroke, be hospitalized for treatments of spontaneous supratentorial intraparenchymal bleedings, be aged 18 years or greater, have non‐surgical treatment for hematoma, be admitted to hospital within poststroke 24 h, and hold premorbid modified Rankin Scale (mRS) score < 2. They would be excluded from this study if patients met one of the exclusion criteria outlined in Figure 1. A group of physical examinees at our hospitals constituted controls. They were not diseased of some chronic sicknesses, e.g., hypertension, diabetes mellitus, and hyperlipidemia, as well as did not present with abnormal values in certain routine tests, such as blood glucose levels, electrolyte levels, leucocyte counts, liver and kidney function, chest X‐ray and electrocardiogram examinations.

### 2.3 Baseline Clinical Data Collections and Assessments of END and Outcome

In three centers, emergency department management, blood pressure, blood glucose and body temperature management, and other conservative therapeutic modalities for hematoma were implemented in compliance with the protocol set forth in the guidelines for the management of ICH (Hemphill et al. 2015). The registered demographical data encompassed age, gender, weight, and height. Body mass index (BMI) was computed on the basis of the formula as follows: weight /height^2^ (kg/m^2^). Vascular risk factors included cigarette smoking, alcohol drinking, hypertension, diabetes mellitus, hyperlipidemia, atrial fibrillation, and coronary heart disease. Specific medications, such as antiplatelet agents, anticoagulants, and statins were recorded. Patients’ neurological statuses were appraised by using the NIHSS (Kwah and Diong 2014). Based on the first‐time head CT scans, hematoma volume was calculated by applying the formula ABC/2 (Kothari et al. 1996), bleeding sites were of two types lobar and deep, and intraventricular or subarachnoidal expansion of hematoma was ascertained. The other recorded information consisted of arterial blood pressure, admission time, and blood sampling time from the onset of stroke symptoms. END was referred to as an increase in the NIHSS score of ≥ 4 or death within the first 24 h after admission (Specogna et al. 2014). Six‐month mRS score of 3–6 following ICH signified a poor outcome (Li et al. 2024).

### 2.4 Blood Collection, Sample Processing, and Immune Analysis

**FIGURE 2:**
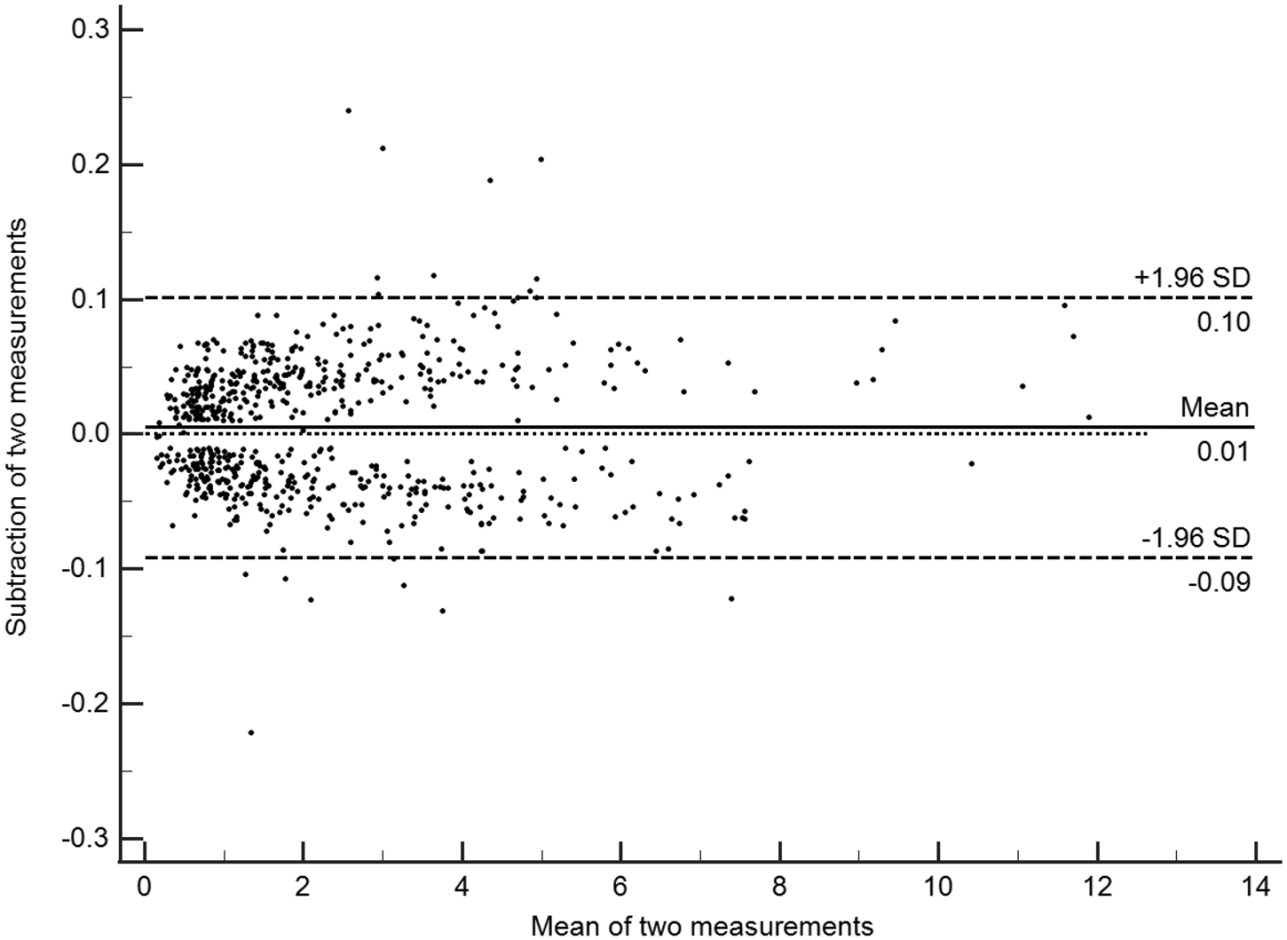
Bland–Altman plot exhibiting accordance between double measurements of serum mixed lineage kinase domain‐like protein levels. There was an excellent accordance between double measurements of serum mixed lineage kinase domain‐like protein levels. SD denotes standard deviation.

Peripheral venous blood was drawn via the median cubital vein upon entry into the study from controls, upon admission from all patients, as well as on days 1, 3, 5, 7, 10, and 15 post‐ICH from some patients. Venous blood was put in 5 mL gel‐containing biochemistry tubes, which were purchased from Hubei New Dashing Material Technology Co., Ltd. (Hubei, China). Following centrifugation at 3000 × *g* for 10 min, serum specimens were isolated and then transferred to Eppendorf tubes (Eppendorf Tubes BioBased, China) for preservation at −80°C condition until final quantification. Thereafter, using a commercially available Enzyme‐Linked Immunosorbent Assay (ELISA) kit (Catalogue number, BES6097K; SHANGHAI BOSEN BIOLOGICAL TECHNOLOGY CO., LTD., China), serum MLKL levels were measured as per the manufacturer's manual. The optical density at a wavelength of 450 nm was read spectrophotometrically using an ELISA reader (Tecan, Infinite M200 pro, Salzburg, Austria). The detection range of the ELISA kit spanned from 31.2 to 2000 pg/mL, with inter‐assay and intra‐assay precision coefficients of variation of ≤ 10 % and 8 % respectively. All quantifications were in duplicate and done in a blinded fashion. The dual measurements were closely correlated (intraclass correlation coefficient = 0.998; *p* < 0.001) and showed good accordance with the Bland–Altman graph (Figure 2). Finally, their mean values were computed for statistical analysis.

### 2.5 Statistical Analysis

The SPSS 19.0 software (SPSS Inc., Chicago, IL, USA) was run for statistical processing. Categorical variables were summarized as frequencies (percentages), and the intergroup comparisons were implemented by adopting the Chi‐Square test or Fisher's exact test as deemed appropriate. The normality test was fulfilled for continuous data. Normally‐distributed data were tested for homogeneity of variance. Means (standard deviations, SDs) and medians (upper‐lower quartiles) were separately reported for parametric and non‐parametric variables. Intergroup distinctions were discerned by employing the independent sample *t*‐test or Mann–Whitney U test as applicable. Using the Kruskal–Wallis test, the disparity in terms of serum MLKL levels was unraveled among several groups. The patients were divided into two groups based on the two outcome variables, that is, poor prognosis and END. Among demographics, vascular risk factors, medication history, clinical indexes, radiological parameters, vital signs, biochemical indicators, and time parameters, some significant factors were revealed by applying univariate analysis. The binary logistic regression analyses, in which significant variables on univariate analysis were incorporated, were employed to investigate the influential factors of the two clinical outcomes. Subgroup analyses were undertaken in order to unveil whether serum MLKL levels interacted with other variables, such as age, gender, BMI, and so forth. Spearman correlation analysis and subsequent multivariate linear regression analysis were performed to determine factors of independent correlation with serum MLKL levels and mRS scores. By applying reliability analysis, the intraclass correlation coefficient was calculated for correlation assessment between two measurements. With the help of MedCalc 9.6.4.0 (MedCalc Software, Mariakerke, Belgium), the receiver operating characteristic (ROC) analysis was carried out and the sample size was computed. The ROC curves were graphed to estimate the predictive efficiency of serum MLKL levels. Using the Bland–Altman plot, the accordance between the two measurements was evaluated. Considering multiple statistical methods, such as intergroup comparisons, ROC curve, multivariate analysis, and bivariate correlation analysis, 161 patients were statistically sufficient here. The GraphPad Prism 9.0 (GraphPad Software, Inc., Boston, MA, USA) was utilized to paint scatter plots, ROC curves, and histograms. The R software (version 3.5.1; https://www.r‐project.org) was deployed to plot restricted cubic spline, nomogram, and calibration curve. A two‐tailed *p* < 0.05 was considered statistically significant.

## 3 Results

### 3.1 Participant Selection and Characteristics

Here, a collective of 227 patients diagnosed with ICH were initially recruited. As outlined in Figure 1, 66 patients were eliminated from this study, and then a total of 161 patients were eligible for final investigation. Among them, 43 suffered from END and 71 experienced a poor six‐month prognosis following ICH. Seventy‐three cases allowed blood collection at several time points. Baseline characteristics were not markedly distinct between all 161 patients and those 73 patients (all *p* > 0.05; Table 1). Also, 73 healthy controls, 40 being males and 33 being females, had a mean age of 57.7 years (SD, 13.5 years) and a mean BMI value of 24.2 kg/m^2^ (SD, 3.5 kg/m^2^). Statistically, gender, age, and BMI did not substantially differ between controls and those 73 patients continuing to provide blood samples at several time points (all *p* > 0.05).

**TABLE 1:** Demographic, clinical, radiological and biochemical data between all patients and those who consented for blood‐drawings at multiple time‐points after acute intracerebral hemorrhage.

Components	All patients	Partial patients	*p* value
Number	161	73	
Demographics			
Age (years)	59.5 ± 11.0	57.9 ± 11.1	0.307
Gender (male/female)	87/74	42/31	0.618
BMI (kg/m^2^)	24.6 ± 3.4	24.7 ± 3.6	0.932
Vascular risk factors			
Cigarette smoking	60 (37.3%)	29 (39.7%)	0.720
Alcohol drinking	55 (34.2%)	20 (27.4%)	0.304
Hypertension	99 (61.5%)	44 (60.3%)	0.860
Diabetes mellitus	34 (21.1%)	12 (16.4%)	0.404
Hyperlipidemia	56 (34.8%)	30 (41.1%)	0.353
Atrial fibrillation	11 (6.8%)	6 (8.2%)	0.705
Coronary heart disease	16 (9.9%)	9 (12.3%)	0.583
Medication history			
Statins	39 (24.2%)	18 (24.7%)	0.943
Anticoagulation drugs	16 (9.9%)	12 (16.4%)	0.156
Antiplatelet drugs	26 (16.2%)	15 (20.6%)	0.412
Clinical indices			
NIHSS score	8 (5–12)	8 (5–11)	0.428
mRS scores			0.956
0	15	8	
1	35	18	
2	40	17	
3	35	12	
4	16	7	
5	13	8	
6	7	3	
Poor prognosis at poststroke six months	71 (44.1%)	30 (41.1%)	0.667
Early neurological deterioration	43 (26.7%)	19 (26.0%)	0.913
Radiological parameters			
Hematoma volume (ml)	13 (8–22)	12 (7–19)	0.411
Intraventricular hemorrhage	37 (23.0%)	10 (13.7%)	0.101
Subarachnoid hemorrhage	15 (9.3%)	5 (6.9%)	0.532
Lobar/deep hematoma	48/113	21/52	0.871
Vital signs			
SAP (mmHg)	143.0 ± 22.2	141.9 ± 22.8	0.471
DAP (mmHg)	85.3 ± 10.3	84.7 ± 10.0	0.580
MAP (mmHg)	104.5 ± 13.8	103.7 ± 13.9	0.532
Laboratory data			
Blood leucocyte count (×10^9^/l)	6.4 (4.8–8.2)	6.2 (4.8–7.8)	0.646
Blood hemoglobin levels (g/l)	126.6 ± 16.1	126.4 ± 17.9	0.932
Blood platelet count (×10^9^/l)	167.0 ± 43.6	164.7 ± 44.2	0.705
Blood glucose levels (mmol/l)	9.7 (7.4–12.7)	9.7 (7.8–14.0)	0.394
Serum MLKL levels at admission (ng/ml)	1.5 (1.0–2.3)	1.6 (1.1–2.4)	0.744
Others			
Admission time (h)	9.4 (5.5–13.5)	9.5 (5.0–13.0)	0.815
Blood‐sampling time (h)	11.3 (6.4–15.5)	10.7 (6.0–15.8)	0.905

*Note*: Data were shown as count (percentage), mean ± standard deviation or median (25th–75th percentiles) as appropriate. Statistical methods included the Chi‐square test, Fisher exact test, Student's 𝑡‐test and Mann–Whitney test. Partial patients refer to those patients who permitted to provide blood sample at several time points.

Abbreviations: BMI, body mass index; DAP, diastolic arterial pressure; MAP, mean arterial pressure; MLKL, mixed lineage kinase domain‐like protein; mRS, modified Rankin scale; NIHSS indicates National Institutes of Health Stroke Scale; SAP, systolic arterial pressure.

### 3.2 Serial Change of Serum MLKL Levels and Its Correlation With Severity

**FIGURE 3:**
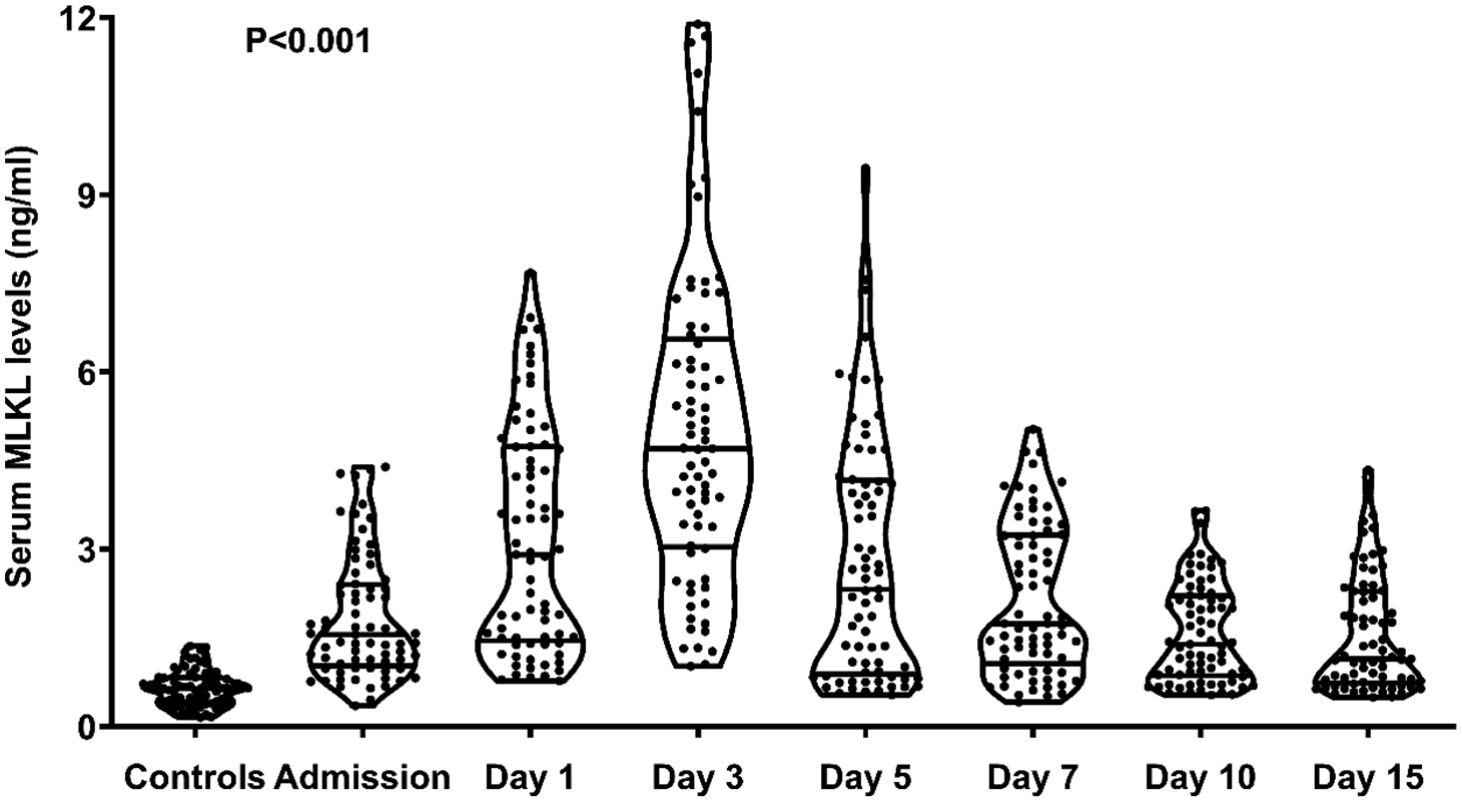
Longitudinal variation of serum mixed lineage kinase domain‐like protein levels subsequent to acute intracerebral hemorrhage. Patients, as opposed to individuals with normal conditions, exhibited notably raised serum mixed lineage kinase domain‐like protein levels during fifteen days after stroke, with peaking value at Day 3 following the onset of stroke (*p* < 0.001). ICH, intracerebral hemorrhage; MLKL, mixed lineage kinase domain‐like protein.

**FIGURE 4:**
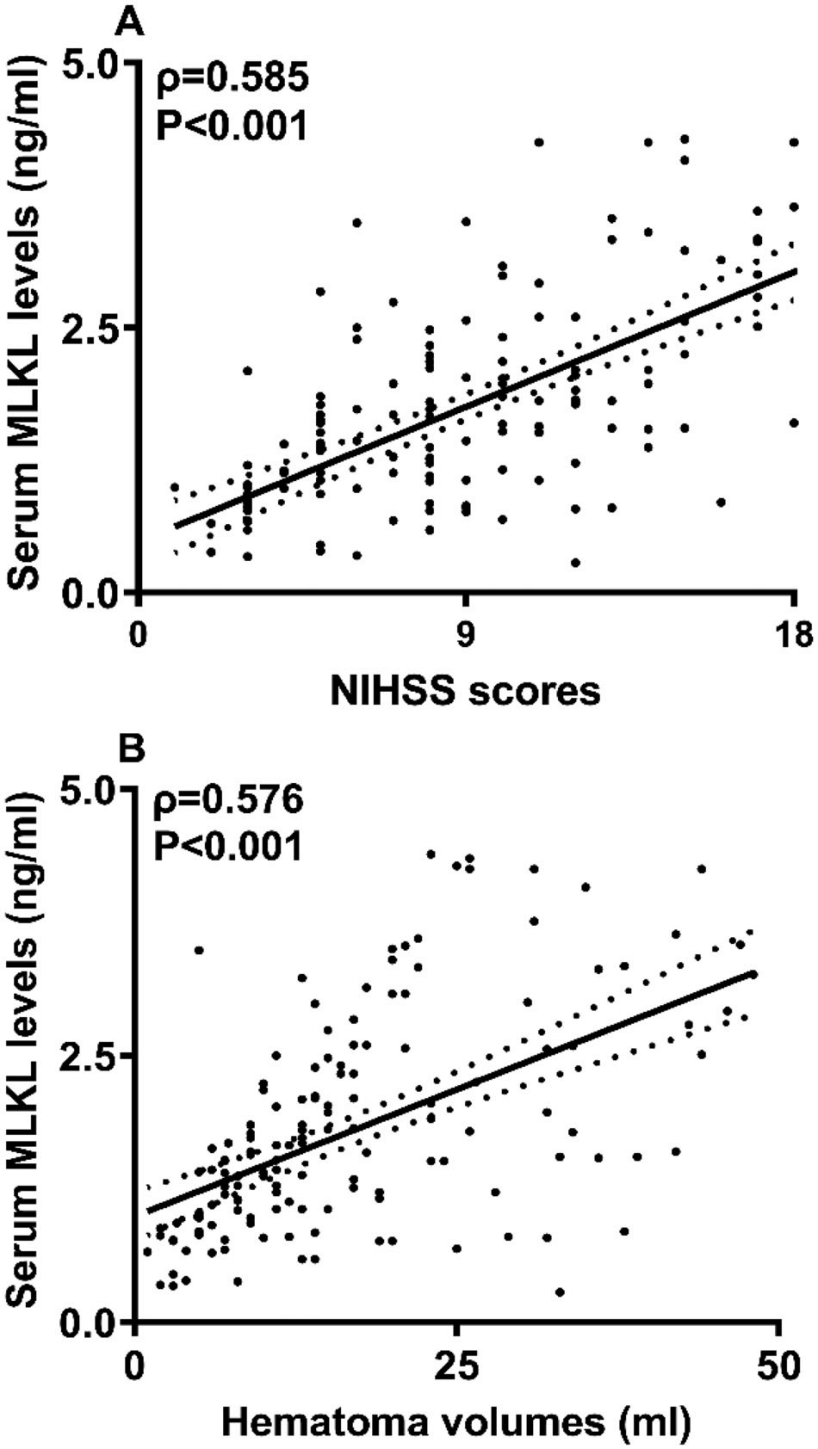
Serum mixed lineage kinase domain‐like protein levels interconnected with severity post‐acute intracerebral hemorrhage. Serum mixed lineage kinase domain‐like protein concentrations were firmly correlated with National Institutes of Health Stroke Scale scores (*p* < 0.001; A) and hematoma volume (*p *< 0.001; B) following intracerebral hemorrhage. MLKL, mixed lineage kinase domain‐like protein; NIHSS, National Institutes of Health Stroke Scale.

Serum MLKL levels were promptly elevated at the admission of those 73 patients, with the peaking levels at Day 3, and were substantially higher during fifteen days than those of controls (*p* < 0.001; Figure 3). Serum MLKL levels were tightly correlated with NIHSS scores (*p* < 0.001; Figure 4A and Table 2), hematoma volume (*p* < 0.001; Figure 4B and Table 2), blood glucose levels (*p* < 0.01; Table 2), and intraventricular hemorrhage (*p* < 0.01; Table 2). By forcing the preceding four factors into the multivariate linear regression model, NIHSS scores (beta, 0.133; 95% CI, 0.088–0.178; variance inflation factor (VIF), 2.326; *p* = 0.011), and hematoma volume (beta, 0.051; 95% CI, 0.037–0.064; VIF, 2.857; *p* = 0.001) were independently correlated with serum MLKL levels.

**TABLE 2:** Demographic, clinical, radiological and biochemical data in correlation with serum mixed lineage kinase domain‐like protein levels and six‐month modified Rank scale scores after acute intracerebral hemorrhage.

Components	Serum MLKL levels		mRS scores	
	*ρ* value	*p* value	*ρ* value	*p* value
Demographics				
Age (years)	0.122	0.122	0.146	0.065
Gender (male/female)	−0.096	0.224	−0.092	0.245
BMI (kg/m^2^)	0.084	0.290	0.043	0.590
Vascular risk factors				
Cigarette smoking	0.103	0.194	−0.064	0.422
Alcohol drinking	0.089	0.260	0.030	0.701
Hypertension	−0.023	0.770	−0.013	0.874
Diabetes mellitus	0.068	0.277	0.181	0.021
Hyperlipidemia	−0.078	0.326	0.084	0.291
Atrial fibrillation	0.033	0.674	0.012	0.881
Coronary heart disease	0.115	0.147	0.011	0.884
Medication history				
Statins	−0.097	0.223	0.107	0.179
Anticoagulation drugs	−0.113	0.154	0.078	0.326
Antiplatelet drugs	−0.139	0.079	0.043	0.588
Clinical severity				
NIHSS score	0.585	< 0.001	0.630	< 0.001
Radiological parameters				
Hematoma volume (mL)	0.576	< 0.001	0.613	< 0.001
Intraventricular hemorrhage	0.218	0.005	0.271	< 0.001
Subarachnoid hemorrhage	0.137	0.082	0.252	0.001
Lobar/deep hematoma	0.006	0.938	−0.032	0.689
Vital signs				
SAP (mmHg)	0.105	0.187	−0.017	0.829
DAP (mmHg)	0.049	0.533	0.019	0.813
MAP (mmHg)	0.081	0.304	−0.002	0.980
Laboratory data				
Blood leucocyte count (× 10^9^/L)	0.127	0.109	0.094	0.236
Blood hemoglobin levels (g/L)	0.002	0.975	0.011	0.888
Blood platelet count (× 10^9^/L)	−0.061	0.444	−0.095	0.231
Blood glucose levels (mmol/L)	0.241	0.002	0.234	0.003
Serum MLKL levels (ng/mL)	1.000	< 0.001	0.577	< 0.001
Others				
Admission time (h)	0.082	0.299	0.017	0.827
Blood‐sampling time (h)	0.107	0.176	0.050	0.530

*Note*: Bivariate correlations were analyzed using the Spearman test.

Abbreviations: BMI, body mass index; DAP, diastolic arterial pressure; END, early neurological deterioration; MAP, mean arterial pressure; MLKL, mixed lineage kinase domain‐like protein; mRS, modified Rankin scale; NIHSS indicates National Institutes of Health Stroke Scale; SAP, systolic arterial pressure.

### 3.3 Serum MLKL Levels and Six‐month mRS Scores Following ICH

**FIGURE 5:**
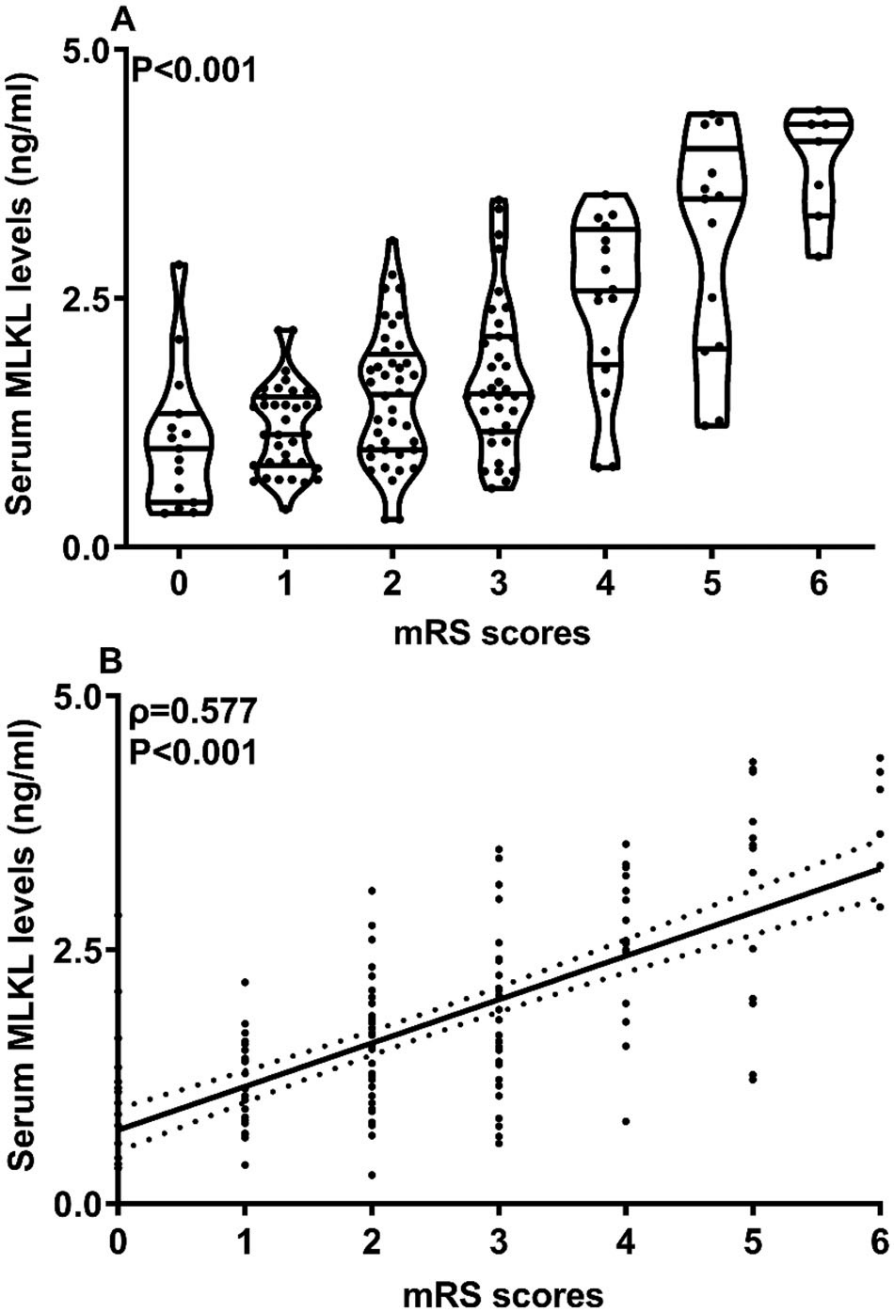
Serum mixed lineage kinase domain‐like protein levels and modified Rankin Scale scores at six months post‐acute intracerebral hemorrhage. Serum mixed lineage kinase domain‐like protein concentrations were obviously lowest in individuals with a modified Rankin Scale score of 0 at six months, progressively increasing across the scores 1 to 5, and peaking among those with a score of 6 (*p* < 0.001; A). And, its levels also displayed a close and positive correlation with six months modified Rankin Scale scores following stroke (*p* < 0.001; B). MLKL, mixed lineage kinase domain‐like protein; mRS, modified Rankin Scale.

Serum MLKL levels were significantly distinct in patients with different mRS scoring gradients, marked by higher levels in parallel to higher scores (*p* < 0.001; Figure 5A) and also displayed a highly positive correlation with continuous mRS scores (*p* < 0.001; Figure 5B). mRS scores were strongly correlated with serum MLKL levels and the other six variables in Table 2 (all *p* < 0.05). With incorporation of the above seven variables into the multivariate linear regression model, the factors of independent relation to mRS were NIHSS scores (beta, 0.141; 95% CI, 0.069–0.214; VIF, 3.077; *p* = 0.011), hematoma volume (beta, 0.033; 95% CI, 0.004–0.061; VIF, 3.682; *p* = 0.014), and serum MLKL levels (beta, 0.707; 95% CI, 0.487–0.927; VIF, 1.742; *p* = 0.023).

### 3.4 Serum MLKL Levels and Six‐month Poor Prognosis Following ICH

**FIGURE 6:**
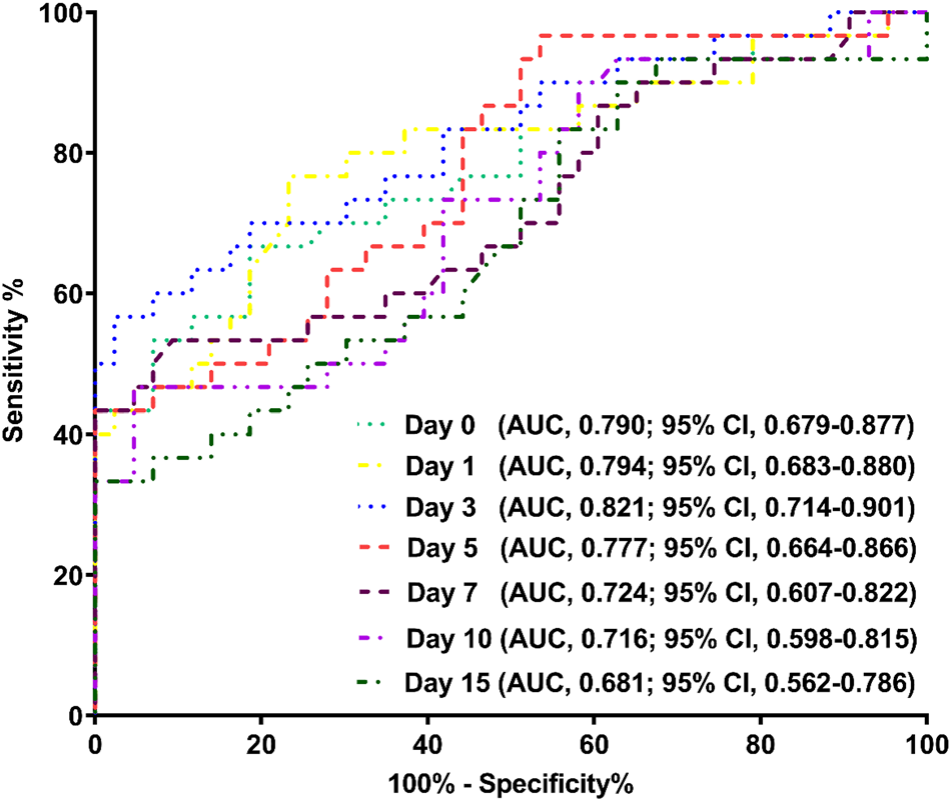
Receiver operating characteristic curve with respect to predictive abilities of different‐time‐point serum mixed lineage kinase domain‐like protein levels on worse neurological outcome at six months post‐acute intracerebral hemorrhage. As opposed to admission serum mixed lineage kinase domain‐like protein levels, those at the other time points had negligibly distinct areas under receiver operating characteristic curve in prognostic prediction (all *p* > 0.05). AUC stands for area under receiver operating characteristic curve; 95% CI, 95% confidence interval.

**FIGURE 7:**
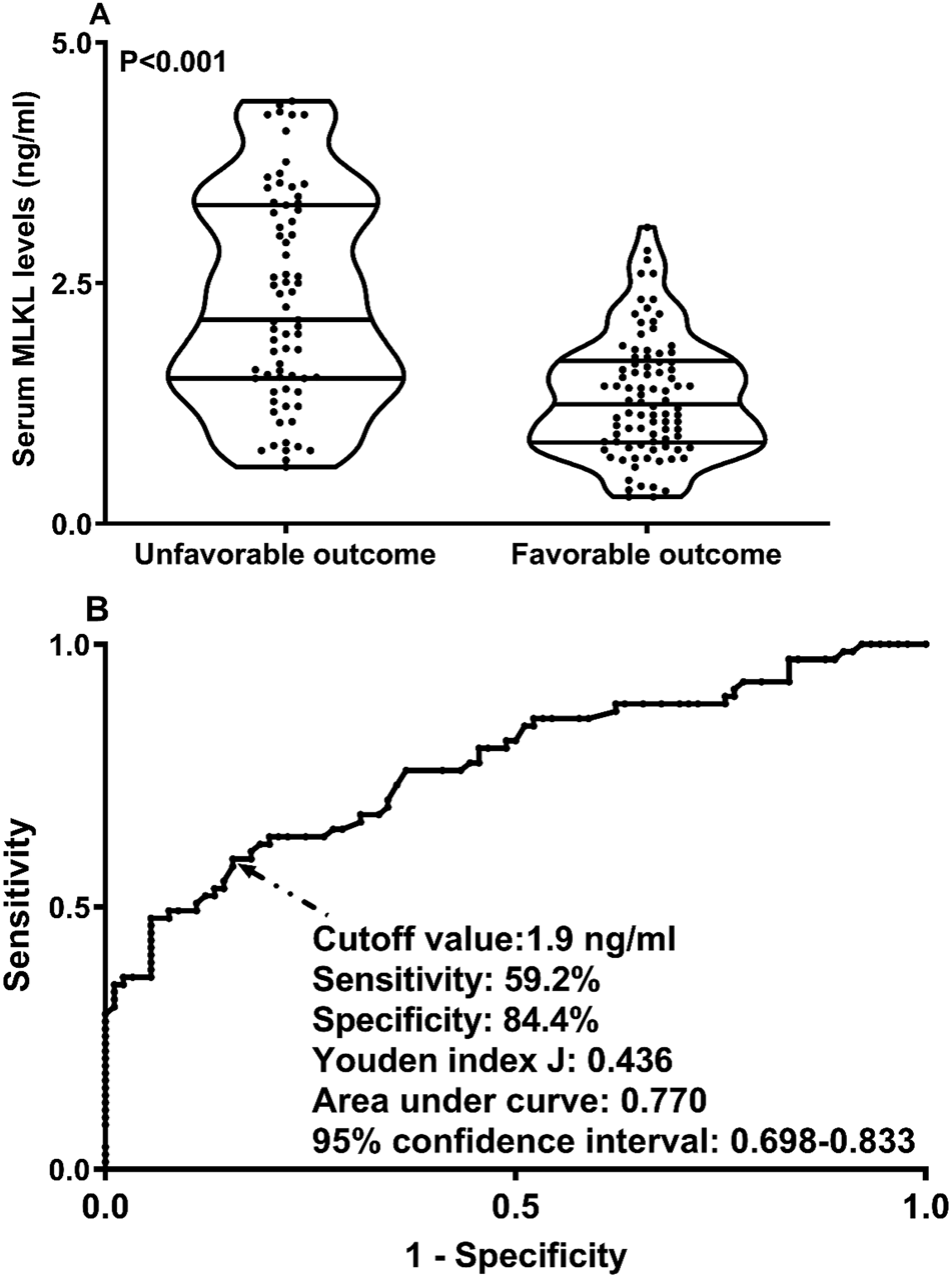
Serum mixed lineage kinase domain‐like protein levels across six‐month neurological outcome after acute intracerebral hemorrhage. Serum mixed lineage kinase domain‐like protein levels were dramatically higher in patients with unfavorable outcome (modified Rankin Scale scores above 2 after stroke) than in those without (p < 0.001; A). Under receiver operating characteristic curve, serum levels of this biomarker could effectively distinguish patients at risk of unfavorable outcome, and the optimal cutoff value was identified with the maximum Youden index for outcome prediction (*p* < 0.001; B). MLKL, mixed lineage kinase domain‐like protein.

**FIGURE 8:**
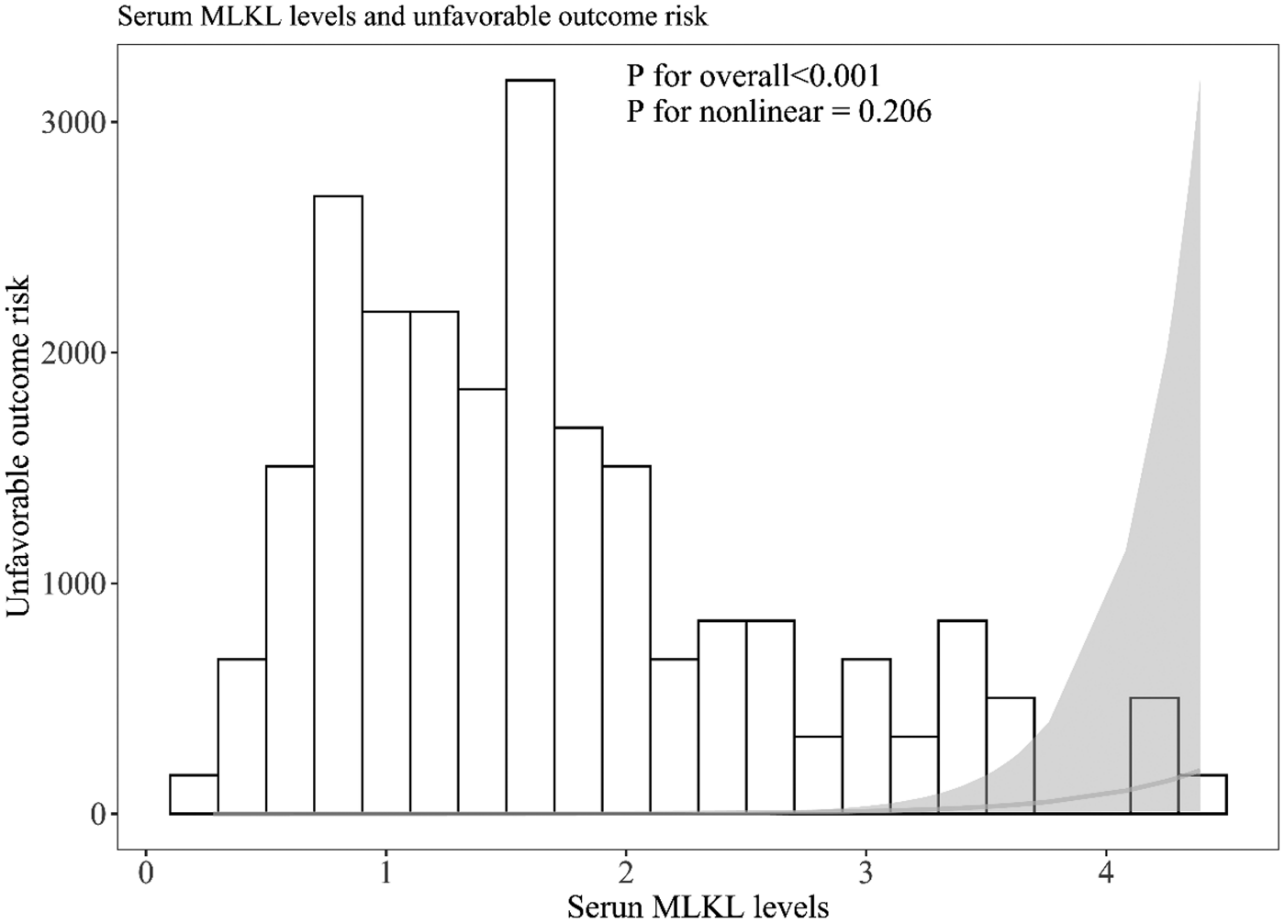
Restricted cubic spline describing the linear correlation of serum mixed lineage kinase domain‐like protein levels with the likelihood of bad outcomes at six months post‐acute intracerebral hemorrhage. Serum mixed lineage kinase domain‐like protein levels were linearly related to the risk of bad prognosis at six months following stroke (*p* for nonlinear > 0.05). MLKL denotes mixed lineage kinase domain‐like protein.

**FIGURE 9:**
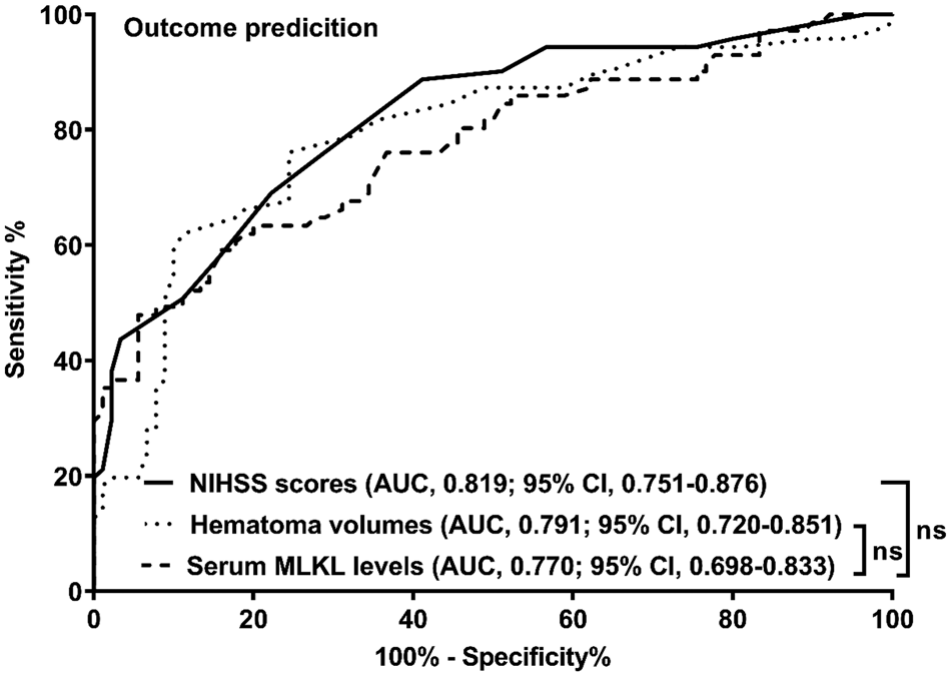
Predictive capability of serum mixed lineage kinase domain‐like protein levels on poor prognosis subsequent to acute intracerebral hemorrhage under the receiver operating characteristic curve. Prognostic ability of serum mixed lineage kinase domain‐like protein levels was equivalent to those of National Institutes of Health Stroke Scale scores and hematoma volume (both *p* > 0.05). AUC, area under curve; 95% CI, 95% confidence interval; MLKL, mixed lineage kinase domain‐like protein; NIHSS, National Institutes of Health Stroke Scale; ns, nonsignificant.

**FIGURE 10:**
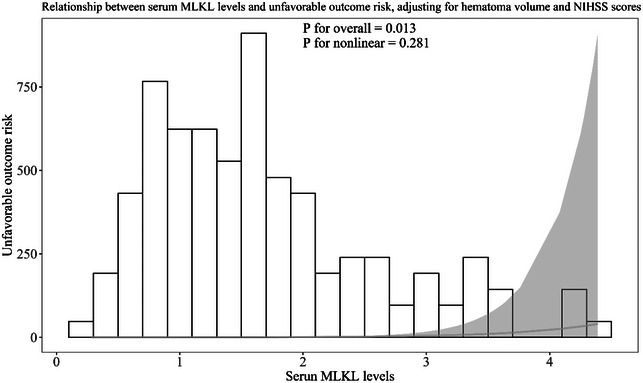
Restricted cubic spline describing the linear correlation of serum mixed lineage kinase domain‐like protein levels with the likelihood of bad outcomes at six months post‐acute intracerebral hemorrhage after adjusting for other confounding factors. Serum mixed lineage kinase domain‐like protein levels had linear relation to the risk of bad prognosis at six months following stroke after adjusting for National Institutes of Health Stroke Scale scores and hematoma volume (*p* for nonlinear > 0.05). MLKL, mixed lineage kinase domain‐like protein; NIHSS, National Institutes of Health Stroke Scale.

**FIGURE 11:**
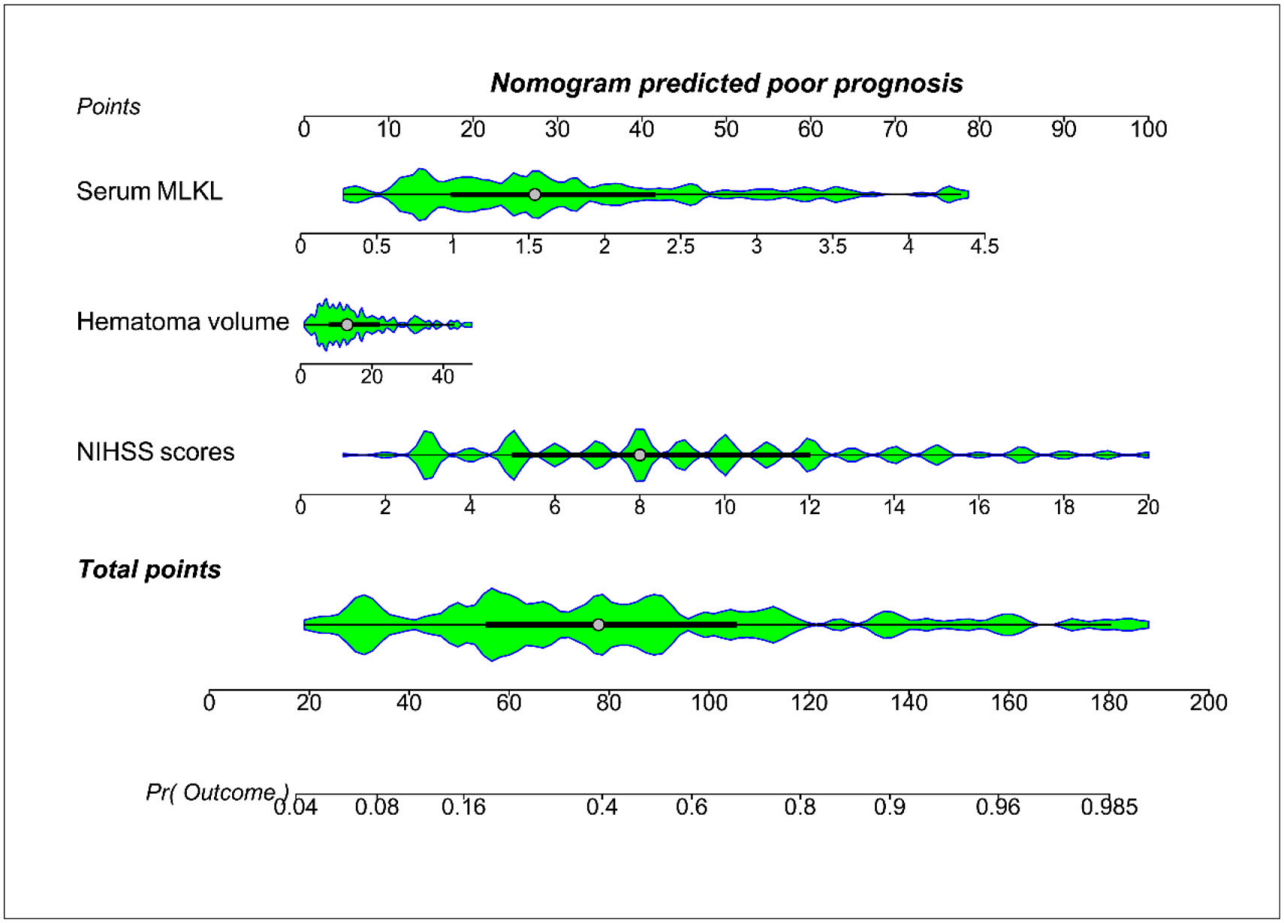
Nomogram evaluating the predictive model for prognosis in individuals diseased of acute intracerebral hemorrhage. Integration of serum mixed lineage kinase domain‐like protein, hematoma size and National Institutes of Health Stroke Scale scores led to establishment of prediction model for differentiating the likelihood of a poor prognosis at six months following acute intracerebral hemorrhage. The model was graphically presented with the nomogram, with different total scores relative to distinct levels of risk. MLKL, mixed lineage kinase domain‐like protein; NIHSS, National Institutes of Health Stroke Scale.

**FIGURE 12:**
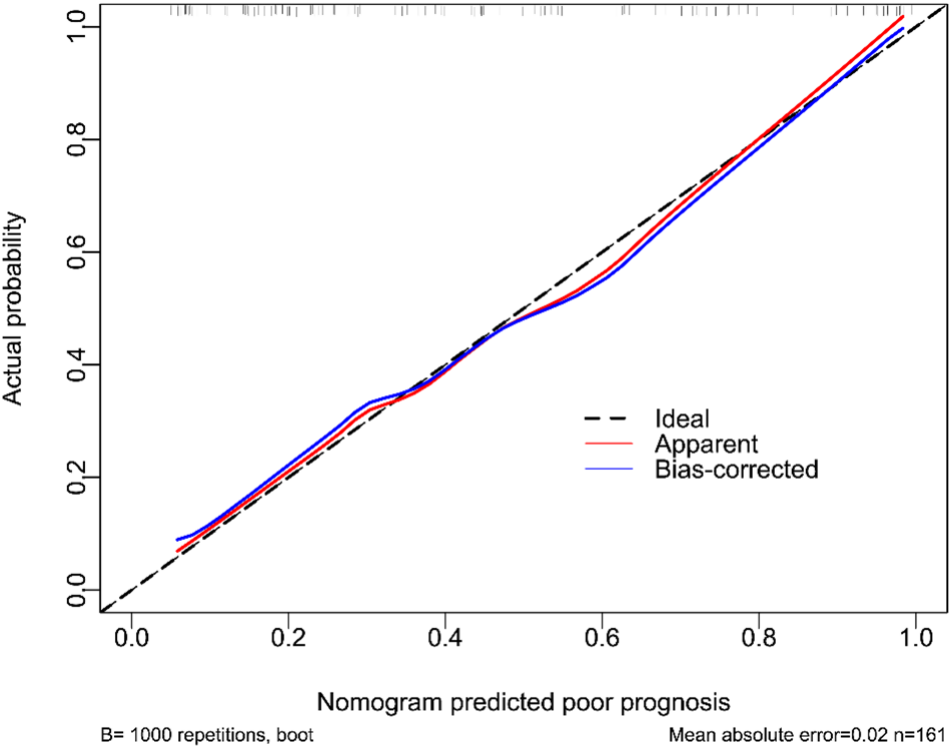
Calibration plot estimating the reliability of the nomogram in predicting the likelihood of a poor prognosis at six months post‐acute intracerebral hemorrhage. The constructed framework kept consistent in forecasting a poor outcome at six months after the onset of acute intracerebral hemorrhage.

Admission serum MLKL levels had AUC of similarity to those at days 1, 3, 5, 7, 10, and 15 following ICH (all *p* > 0.05; Figure 6). Significantly higher serum MLKL levels were seen in patients with poor prognosis than in those presenting with good prognosis (*p* < 0.001; Figure 7A). Also, poor prognosis possibility was efficaciously distinguished by serum MLKL levels, and 1.9 ng/mL was identified as a threshold with the Youden method (Figure 7B). Linear relation of serum MLKL levels to poor prognosis probability was demonstrated (*p* for nonlinear >0.05; Figure 8). Serum MLKL levels and the other six factors in Table 3 were substantially different between patients with poor prognosis and those with good prognosis (all *p* < 0.05). Following entry of all significantly distinct variables into the binary logistic regression model, it was revealed that NIHSS scores (OR, 1.208; 95% CI, 1.011–1.445; *p* = 0.008), hematoma volume (OR, 1.077; 95% CI, 1.017–1.141; *p* = 0.012), and serum MLKL levels (OR, 2.286; 95% CI, 1.324–3.946; *p* = 0.038) independently forecasted six‐month poor prognosis subsequent to ICH. Non‐interactions were found between serum MLKL levels and other variables, such as age, gender, and more (all *p* > 0.05; Table 4). As depicted in Figure 9, the predictive ability of serum MLKL levels resembled those of NIHSS scores and hematoma volumes (both *p* > 0.05). Even if adjusting for NIHSS scores and hematoma volume, serum MLKL levels were still linearly correlated with the risk of poor prognosis (*p* for nonlinear > 0.05; Figure 10). Moreover, the prediction model with the consolidation of serum MLKL levels, NIHSS scores, and hematoma volume was visualized via a nomogram (Figure 11) and had good stability (Figure 12).

**TABLE 3:** Demographic, clinical, radiological and biochemical data in association with early neurologic deterioration and six‐month poor prognosis after acute intracerebral hemorrhage.

Components	Neurological function outcome			Early neurological deterioration		
	Poor prognosis	Good prognosis	*p* value	Presence	Absence	*p* value
Demographics						
Age (years)	60.6 ± 11.4	58.6 ± 10.7	0.239	57.9 ± 12.4	60.1 ± 10.5	0.320
Gender (male/female)	34/37	53/37	0.164	23/20	64/54	0.933
BMI (kg/m^2^)	24.6 ± 2.9	24.7 ± 3.7	0.974	24.5 ± 2.9	24.7 ± 3.5	0.725
Vascular risk factors						
Cigarette smoking	22 (31.0%)	38 (42.2%)	0.143	21 (48.8%)	39 (33.1%)	0.067
Alcohol drinking	30 (42.3%)	25 (27.8%)	0.054	14 (32.6%)	41 (34.8%)	0.796
Hypertension	41 (57.8%)	58 (64.4%)	0.386	25 (58.1%)	74 (62.7%)	0.598
Diabetes mellitus	22 (31.0%)	12 (13.3%)	0.006	13 (30.2%)	21 (17.8%)	0.087
Hyperlipidemia	27 (38.0%)	29 (32.2%)	0.443	17 (39.5%)	39 (33.1%)	0.445
Atrial fibrillation	5 (7.0%)	6 (6.7%)	0.925	4 (9.3%)	7 (5.9%)	0.486
Coronary heart disease	8 (11.3%)	8 (8.9%)	0.616	3 (7.0%)	13 (11.0%)	0.562
Medication history						
Statins	20 (28.2%)	19 (21.1%)	0.299	11 (25.6%)	28 (23.7%)	0.808
Anticoagulation drugs	10 (14.1%)	6 (6.7%)	0.118	4 (9.3%)	12 (10.2%)	1.000
Antiplatelet drugs	13 (18.3%)	13 (14.4%)	0.508	4 (9.3%)	22 (18.6%)	0.154
Clinical severity						
NIHSS score	12 (9–15)	7 (5–9)	< 0.001	13 (9–17)	8 (5–10)	< 0.001
Radiological parameters						
Hematoma volume (mL)	21 (14–31)	9 (6–13)	< 0.001	22 (14–36)	11 (7–17)	< 0.001
Intraventricular hemorrhage	23 (32.4%)	14 (15.6%)	0.012	16 (37.2%)	21 (17.8%)	0.010
Subarachnoid hemorrhage	12 (16.9%)	3 (3.3%)	0.003	8 (18.6%)	7 (5.9%)	0.027
Lobar/deep hematoma	23/48	25/65	0.525	15/28	33/85	0.396
Vital signs						
SAP (mmHg)	144.1 ± 22.4	142.1 ± 22.2	0.579	144.3 ± 21.0	142.6 ± 22.8	0.664
DAP (mmHg)	85.5 ± 10.9	85.1 ± 9.9	0.785	86.7 ± 11.8	84.8 ± 9.7	0.308
MAP (mmHg)	105.1 ± 14.3	104.1 ± 13.5	0.664	105.9 ± 14.2	104.1 ± 13.7	0.459
Laboratory data						
Blood leucocyte count (×10^9^/L)	6.7 (4.9–8.5)	6.3 (4.7–7.8)	0.346	5.9 (4.7–8.1)	6.7 (5.0–8.2)	0.737
Blood hemoglobin levels (g/L)	126.0 ± 16.2	127.0 ± 16.1	0.681	130.1 ± 15.2	125.3 ± 16.3	0.090
Blood platelet count (×10^9^/L)	160.8 ± 38.8	171.9 ± 46.7	0.111	168.5 ± 32.5	166.5 ± 47.2	0.792
Blood glucose levels (mmol/L)	10.8 (7.6–14.2)	9.0 (7.4–11.0)	0.026	10.8 (8.0–13.7)	9.2 (7.2–12.5)	0.070
Serum MLKL levels (ng/mL)	2.1 (1.5–3.3)	1.2 (0.9–1.7)	< 0.001	2.5 (1.6–3.5)	1.4 (0.9–1.9)	<0.001
Others						
Admission time (h)	9.8 (5.9–14.5)	9.3 (4.5–12.8)	0.224	9.6 (5.0–13.3)	8.8 (5.8–13.7)	0.974
Blood‐sampling time (h)	12.1 (7.7–16.8)	10.8 (5.3–14.8)	0.118	11.4 (7.1–15.9)	11.0 (5.4–15.2)	0.572

*Note*: Data were shown as count (percentage), mean ± standard deviation or median (25th–75th percentiles) as appropriate. Statistical methods included the Chi‐square test, Fisher exact test, Student's 𝑡‐test and Mann–Whitney test.

Abbreviations: BMI, body mass index; DAP, diastolic arterial pressure; END, early neurological deterioration; MAP, mean arterial pressure; MLKL, mixed lineage kinase domain‐like protein; NIHSS indicates National Institutes of Health Stroke Scale; SAP, systolic arterial pressure.

**TABLE 4:** Subgroup analysis for the associations of serum mixed lineage kinase domain‐like protein levels with 6‐month poor prognosis and early neurological deterioration after acute intracerebral hemorrhage and interaction analyses between subgroups.

Subgroup analysis	Number	Six‐month poor prognosis			Early neurological deterioration		
		OR (95% CI)	*p* value	*P*_interaction_ value	OR (95% CI)	*p* value	*P*_interaction_ value
Age							
≥ 65 years	60	1.269 (0.905–1.780)	0.168	0.214	1.284 (0.551–2.993)	0.562	0.264
< 65 years	101	2.865 (1.343–6.112)	0.006		3.179 (1.616–6.255)	0.001	
Gender							
Male	87	3.788 (1.600–8.965)	0.002	0.246	1.850 (1.016–3.369)	0.044	0.718
Female	74	1.249 (0.574–2.731)	0.278		1.843 (0.963–3.529)	0.065	
Body mass index							
> 24 kg/m^2^	90	2.189 (1.080–4.438)	0.030	0.618	1.919 (1.054–3.495)	0.033	0.810
≤ 24 kg/m^2^	71	3.048 (0.956–4.390)	0.065		2.156 (1.083–4.290)	0.029	
Hypertension							
Yes	99	2.706 (1.417–5.168)	0.013	0.640	1.935 (1.124–3.332)	0.017	0.371
No	62	2.109 (1.162–4.588)	0.017		1.791 (0.864–3.712)	0.117	
Diabetes mellitus							
Yes	34	4.281 (0.883–20.747)	0.071	0.603	1.544 (0.709–3.361)	0.274	0.216
No	127	2.262 (1.217–4.203)	0.010		2.070 (1.211–3.537)	0.008	
Hyperlipidemia							
Yes	56	2.309 (0.820–6.505)	0.113	0.476	1.729 (0.810–3.691)	0.157	0.491
No	105	2.411 (1.230–4.726)	0.010		2.101 (1.179–3.744)	0.012	
Cigarette consumption							
Yes	60	1.382 (0.558–3.424)	0.484	0.205	1.572 (0.895–2.758)	0.115	0.398
No	101	12.732 (2.224–72.910)	0.004		2.541 (1.230–5.248)	0.012	
Alcohol consumption							
Yes	55	1.113 (0.379–3.274)	0.845	0.174	1.626 (0.718–3.682)	0.244	0.196
No	106	3.188 (1.494–6.799)	0.003		2.046 (1.193–3.510)	0.009	
Atrial fibrillation							
Yes	11	—	—	0.356	—	—	0.103
No	150	2.255 (1.282–3.967)	0.005		1.713 (1.085–2.704)	0.021	
Coronary heart disease							
Yes	16	—	—	0.292	—	—	0.301
No	145	2.425 (1.289–4.559)	0.006		1.925 (1.196–3.096)	0.007	
Previous statin use							
Yes	39	1.592 (0.430–5.888)	0.486	0.323	4.841 (1.000–23.430)	0.050	0.592
No	122	2.400 (1.213–4.750)	0.012		1.785 (1.069–2.981)	0.027	
Previous anticoagulant use							
Yes	16	—	—	0.143	—	—	0.122
No	145	2.755 (1.487–5.106)	0.001		1.889 (1.171–3.046)	0.009	
Previous antiplatelet use							
Yes	26	—	—	0.059	—	—	0.123
No	135	2.533 (1.407–4.562)	0.002		1.953 (1.179–3.235)	0.009	

OR indicates odds ratio; 95% CI, 95% confidence interval.

### 3.5 Serum MLKL Levels and END Following ICH

**FIGURE 13:**
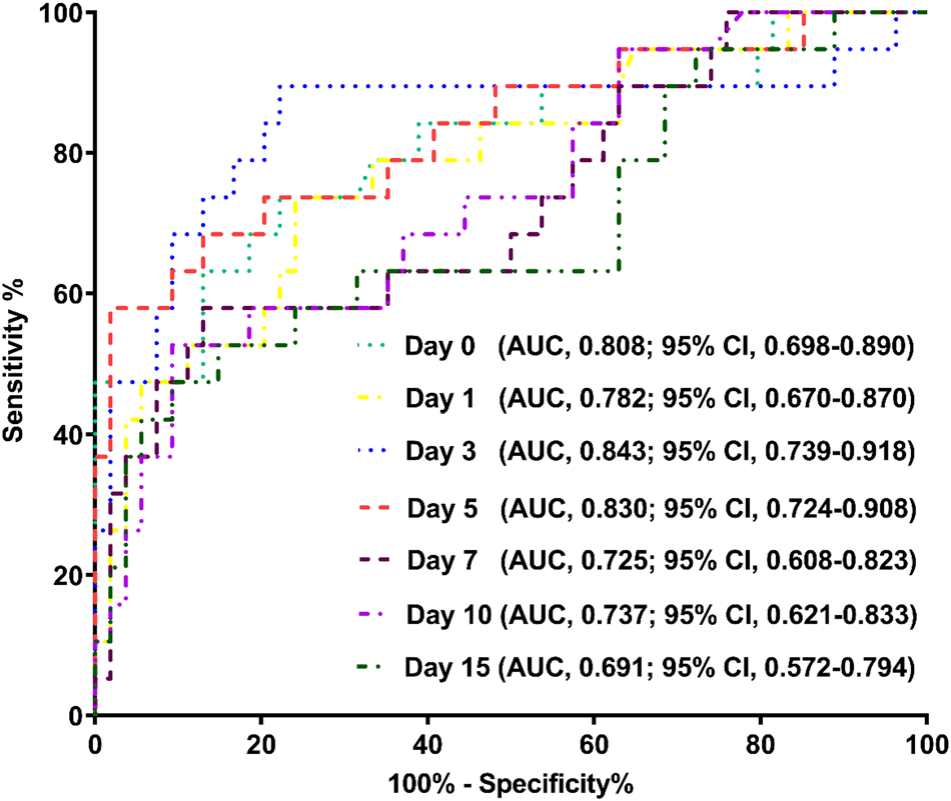
Receiver operating characteristic curve displaying predictive capabilities of serum mixed lineage kinase domain‐like protein levels at several time points on early neurological deterioration after acute intracerebral hemorrhage. There were non‐significant differences in terms of predictive ability for early neurological deterioration between serum mixed lineage kinase domain‐like protein levels at admission (Day 0) and at the other time points under receiver operating characteristic curve (all *p* > 0.05). AUC, area under receiver operating characteristic curve; 95% CI, 95% confidence interval.

**FIGURE 14:**
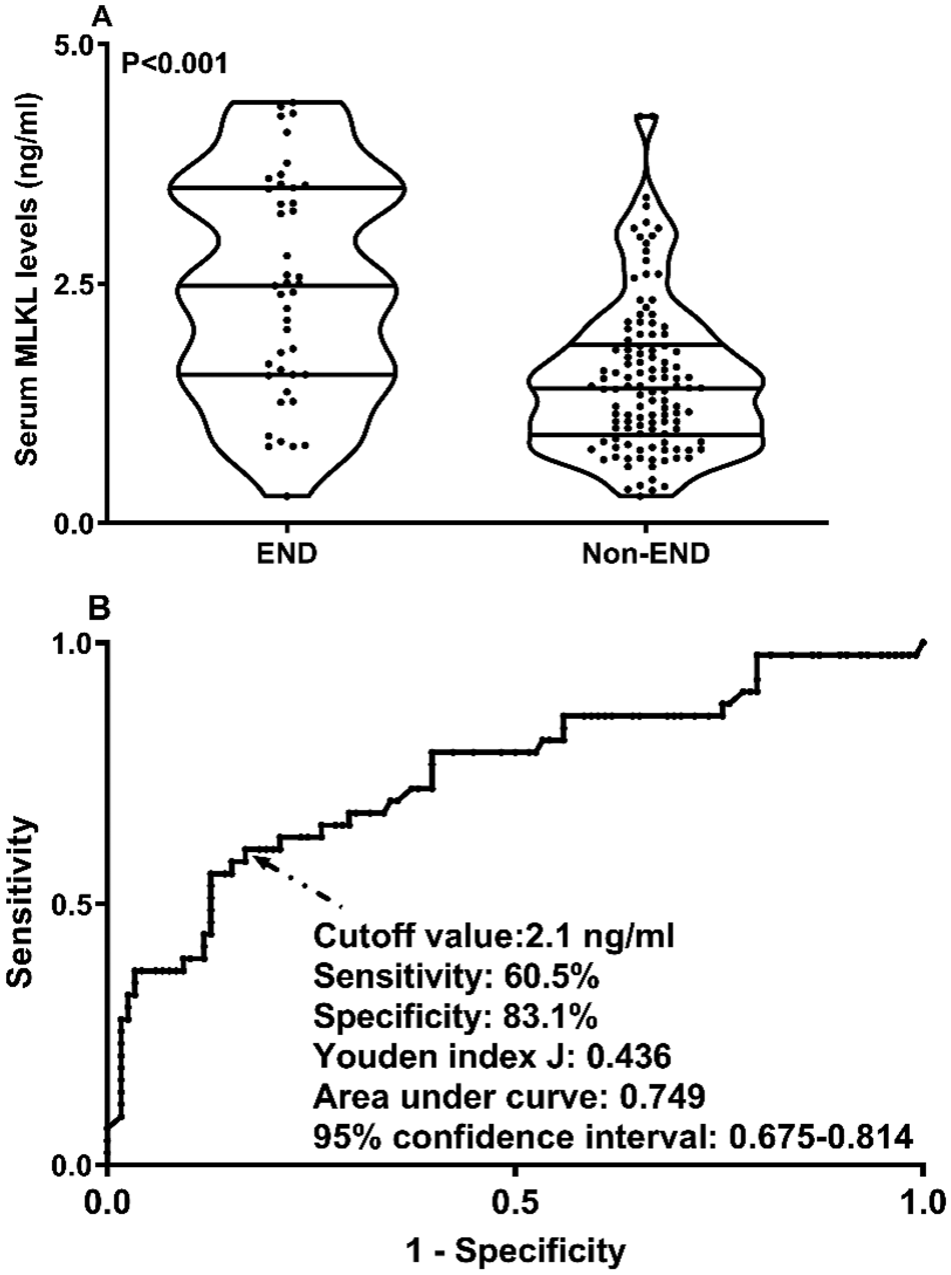
Serum mixed lineage kinase domain‐like protein levels by early neurological deterioration subsequent to acute intracerebral hemorrhage. Patients with early neurological deterioration had markedly higher serum mixed lineage kinase domain‐like protein levels than the other remainders (*p* < 0.001; A). Under receiver operating characteristic curve, its serum levels could efficiently discriminate likelihood of early neurological deterioration and the optimal cutoff value was chosen with the maximum Youden index for predicting early neurological deterioration (*p* < 0.001; B). END, early neurological deterioration; MLKL, mixed lineage kinase domain‐like protein.

**FIGURE 15:**
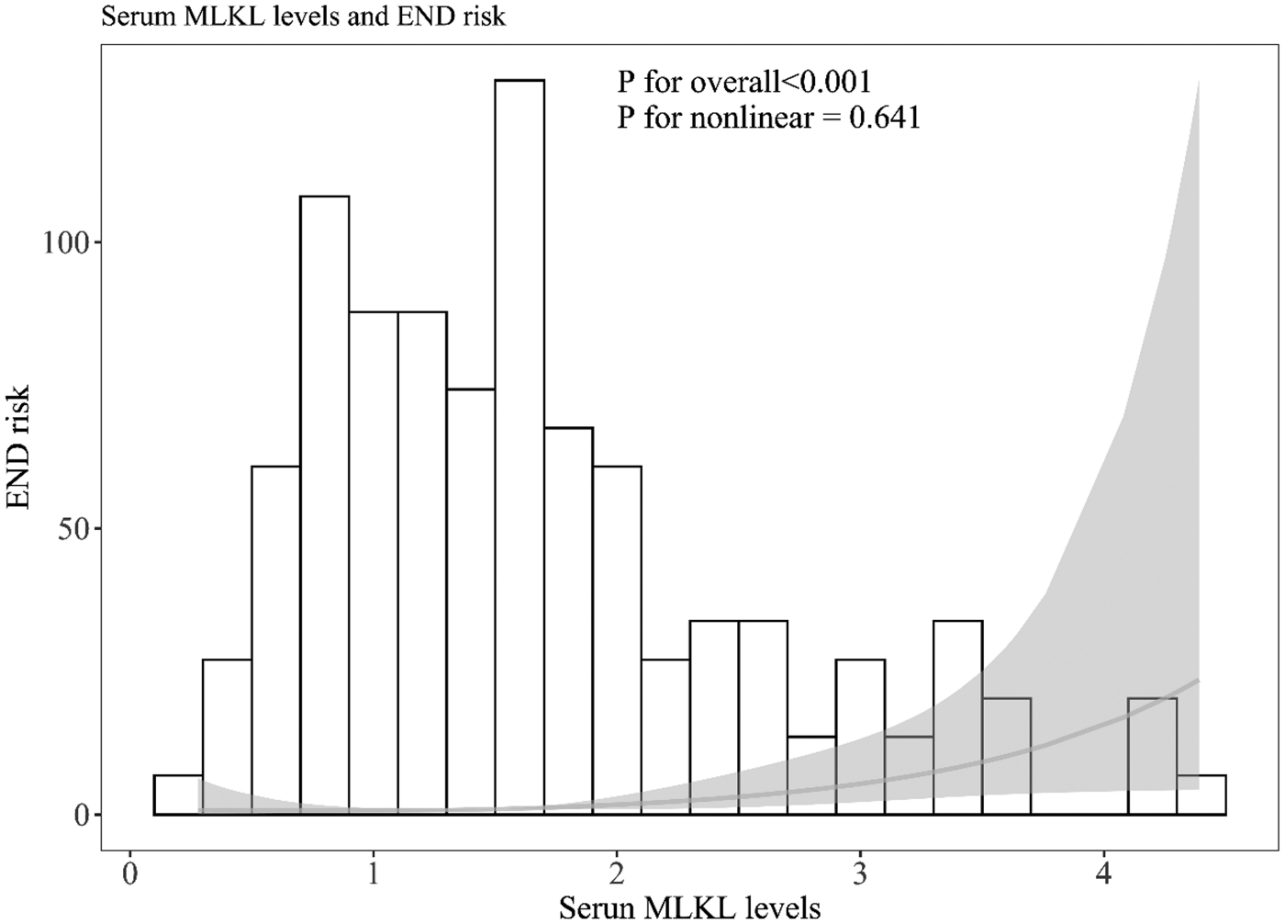
Restricted cubic spline outlining the linear relationship between serum mixed lineage kinase domain‐like protein levels and the likelihood of early neurological deterioration following acute intracerebral hemorrhage. A linear correlation was revealed between serum mixed lineage kinase domain‐like protein levels and probability of early neurological deterioration after acute brain hemorrhage (*p* for nonlinear > 0.05). END, early neurological deterioration; MLKL denotes mixed lineage kinase domain‐like protein.

**FIGURE 16:**
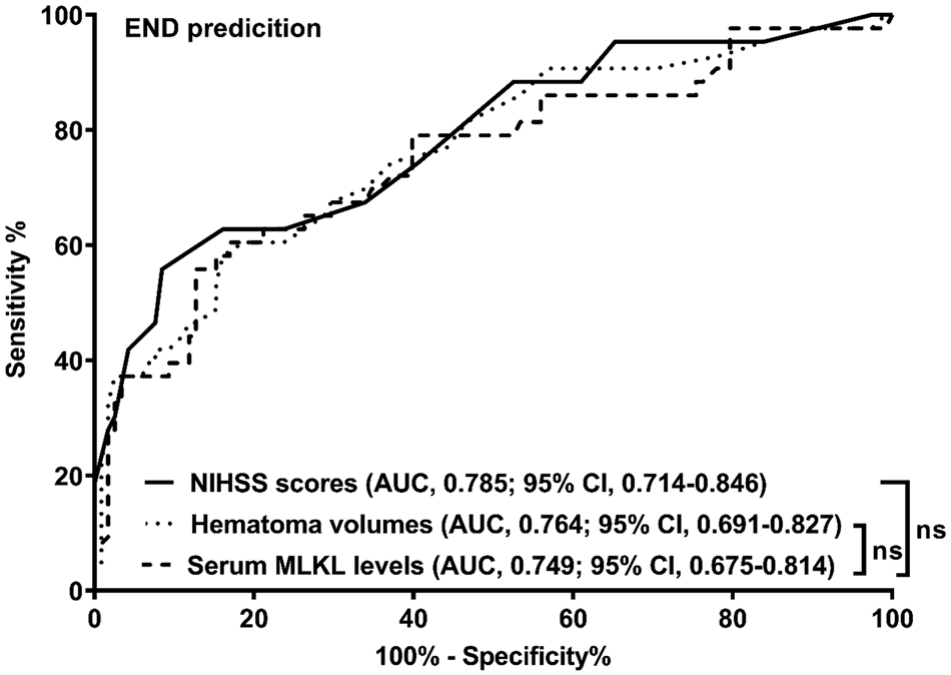
Predictive performance of serum mixed lineage kinase domain‐like protein levels on early neurological deterioration following acute intracerebral hemorrhage using receiver operating characteristic curve analysis. Serum mixed lineage kinase domain‐like protein concentrations took possession of analogous predictive ability on early neurological deterioration, as compare to National Institutes of Health Stroke Scale scores and hematoma volume (both *p* > 0.05). AUC, area under curve; 95% CI, 95% confidence interval; END, early neurological deterioration; MLKL, mixed lineage kinase domain‐like protein; NIHSS, National Institutes of Health Stroke Scale; ns, nonsignificant.

**FIGURE 17:**
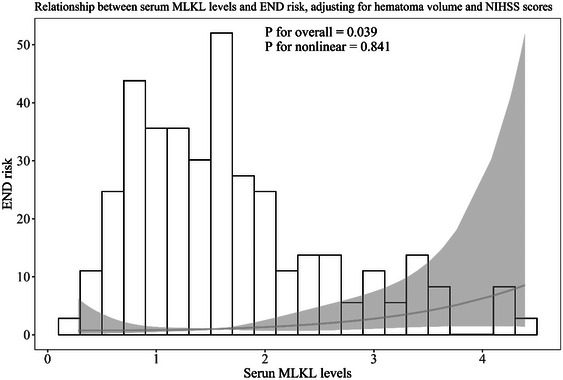
Restricted cubic spline showing the linear relationship serum mixed lineage kinase domain‐like protein levels and the risk of early neurological deterioration post‐acute intracerebral hemorrhage after adjusting for other confounding factors. Serum mixed lineage kinase domain‐like protein concentrations were linearly related to the risk of early neurological deterioration following stroke after adjusting for National Institutes of Health Stroke Scale scores and hematoma volume (*p* for nonlinear > 0.05). END, early neurological deterioration; MLKL, mixed lineage kinase domain‐like protein; NIHSS, National Institutes of Health Stroke Scale.

**FIGURE 18:**
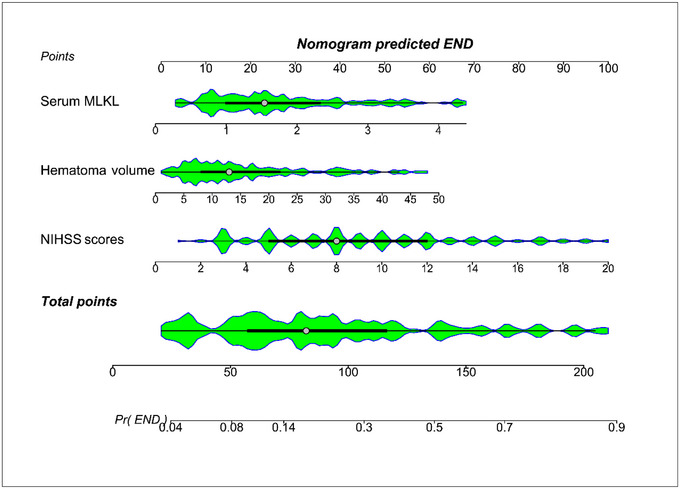
Nomogram estimating the predictive model for early neurological deterioration following acute intracerebral hemorrhage. A model, in which serum mixed lineage kinase domain‐like protein, hematoma volume and National Institutes of Health Stroke Scale scores were merged, was constructed to predict early neurological deterioration following acute intracerebral hemorrhage. The nomogram visually reflected the model, with different total scores corresponding to distinct levels of risk. END, early neurological deterioration; MLKL, mixed lineage kinase domain‐like protein; NIHSS, National Institutes of Health Stroke Scale.

**FIGURE 19:**
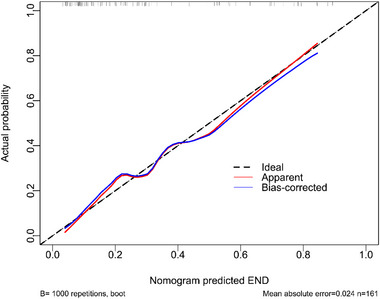
Calibration curve assessing reliability of the nomogram for distinguishing early neurological deterioration post‐acute intracerebral hemorrhage. The established framework was consistent in anticipating early neurological deterioration following acute intracerebral hemorrhage. END, early neurological deterioration.

AUC of admission serum MLKL levels numerically, but not statistically significantly differed from those on days 1, 3, 5, 7, 10, and 15 (all *p* > 0.05; Figure 13). Patients with END development, in contrast to those without, had substantially higher serum MLKL levels at admission (*p* < 0.001; Figure 14A). Alternatively, serum MLKL levels held high discrimination efficiency on END risk and its level above 2.1 ng/mL was predictive of END with the maximum Youden index (Figure 14B). Statistically, there was a linear relationship between serum MLKL levels and END risk (*p* for nonlinear > 0.05; Figure 15). Aside from serum MLKL levels, the other four parameters in Table 3 were significantly connected with END (all *p* < 0.05). The addition of all five parameters in a multiple factorial model led to the finding that hematoma volume (OR, 1.064; 95% CI, 1.012–1.120; *p* = 0.012) and serum MLKL levels (OR, 1.902; 95% CI, 1.229–2.945; *p* = 0.014) were independent associated with END. Serum MLKL levels had non‐significant interaction with age, gender, hypertension, etc. (all *p* > 0.05; Table 4). In addition, no differences in terms of AUC for predicting END were found when serum MLKL levels were compared to NIHSS scores and hematoma volumes (both *p* > 0.05; Figure 16). After adjusting for NIHSS scores and hematoma volume, the linear relationship still existed between serum MLKL levels and END risk (*p* for nonlinear > 0.05; Figure 17). Moreover, serum MLKL, NIHSS scores, and hematoma volume were put together to construct an END prediction model of representation by a nomogram (Figure 18), and the model presented with a good steadiness (Figure 19).

## 4 Discussion

To the best of our knowledge, it keeps unclear about circulating MLKL levels after acute ICH. We measured serum MLKL levels in patients with ICH and individuals with normal conditions, and subsequently made a substantial discovery of the enhancement of serum MLKL levels at admission until Day 15 after ICH, as compared to controls, with the significantly highest levels at Day 3. Moreover, post‐ICH serum MLKL levels showed an independent correlation with NIHSS scores, hematoma volume, and poststroke six‐month mRS scores. Noteworthily, Serum MLKL levels, alongside NIHSS scores and hematoma volume, were independently associated with END and poor prognosis six months after ICH. However, the linearity relations were existent, while interactions with some common variables were not affirmed. In addition, as for predicting END and poor prognosis following acute ICH, serum MLKL levels at admission presented with analogous AUC as those at other time‐points among those seventy‐three patients, and the results were consistent when compared to NIHSS scores and hematoma volume in all patients. In summary, serum MLKL may appear as an encouraging factor in prognosticating END and poor prognosis after ICH.

Definitely, MLKL, a paramount component of necroptosis, can be expressed in brain tissues under physiological circumstances (Nakano 2024). Moreover, its expressions by astrocytes and neurons were markedly raised in experimental brain injury (Yuan et al. 2023; Huang et al. 2022; Lule et al. 2021; Zhou et al. 2017). Undoubtedly, it may be a detrimental factor because its inhibition or gene deletion could extremely lessen brain edema, improve the permeability of the blood‐brain barrier, reduce neuronal apoptosis, and diminish neurological deficits in animal experiments (Yuan et al. 2023; Huang et al. 2022; Lule et al. 2021; Zhou et al. 2017). Hence, MLKL has become a therapeutic target for acute brain injury diseases.

In both participants with normal conditions and patients diagnosed with ICH in this study, serum MLKL levels were in duplicate measured, and subsequently mean values of the double measurements were applied for final data analysis. Using reliability analysis and plotting the Bland–Altman graph, an excellent accordance was confirmed between the two measurements, meaning the data should be reliable and steady. Afterward, for the sake of investigating the temporal change of serum MLKL levels after acute ICH, a group of 73 patients voluntarily provided blood samples at multiple time points following ICH. This group of patients had analogous baseline features as all patients, signifying this fraction of patients may be accurately representative of the whole cohort of patients. As expected, serum MLKL levels non‐statistically significantly differed between the two groups of patients. Clearly, serum MLKL levels were substantially elevated during fifteen days, as compared to controls; moreover, its highest levels occurred at Day 3 after stroke. The data give strong support to the conception that blood MLKL levels should be accepted as an elevation following ICH. Given that MLKL could be expressed dramatically in injured brain tissues, with the implicit possession of detrimental effects in acute brain injury (Yuan et al. 2023; Huang et al. 2022; Lule et al. 2021; Zhou et al. 2017), it is inferred that production of MLKL from the central nervous system and subsequent releasing may contribute to increment of MLKL levels in the peripheral blood of ICH patients.

NIHSS and hematoma volume are the two conventional severity indicators of ICH (Geng et al. 2024; J. Wu, et al. 2024; Yu et al. 2023). Here, serum MLKL levels were closely correlated with them using the multivariate linear regression analysis. Although there have not been other reports about the relationship between serum MLKL levels and the severity of ICH, this study can offer pilot evidence to deduce that serum MLKL may be recommended as a predictor for mirroring stroke severity following ICH.

In the current study, serum MLKL levels at the other time points did not display significant advantages over those at admission in terms of AUCs for predicting END or poor six‐month prognosis after ICH. In other words, serum MLKL levels had steady predictive ability within at least 15 days regardless of blood‐collection time. In consideration of a larger sample size, as the total of 163 patients over those 73 patients, the 163 patients were considered as the subjects for analyzing its association with both END and poor prognosis following ICH. Clearly, NIHSS and hematoma volume are very frequently demonstrated to be the two determinants of both END and poor prognosis of ICH (Shentu et al. 2023; Li, Shan, et al. 2023; C. Zhang et al. 2023; Li, Lv, et al. 2023). Nevertheless, NIHSS was not an independent predictor of END here. Admittedly, a small sample number of positive END cases may be an explanation. Intriguingly, serum MLKL levels at admission were revealed to be independently associated with both END and poor prognosis six months after ICH. Hence, serum MLKL is believed to hold the chance to serve as a potential predictor of END and poor prognosis in ICH.

Here, ample statistical methods were done to deeply determine the relation of serum MLKL levels to END and poor prognosis of ICH patients. Specifically, even if adjusting for NIHSS scores and hematoma volume, serum MLKL levels were still linearly correlated with END and poor prognosis six months after ICH. In addition, serum MLKL levels non‐significantly interacted with other variables, such as age, gender, hypertension, etc. Also, serum MLKL levels owned similar predictive ability in terms of AUC, as compared to NIHSS scores and hematoma volume. Furthermore, the models of END and poor prognosis were built, in which NIHSS scores, hematoma volume, and serum MLKL were merged. The models were visually described using the nomograms and performed stably. Taken together, serum MLKL may potentially contribute to the prognostic prediction of ICH.

Neuroinflammation, a critical mechanism of various pathophysiological processes in neurological diseases, is firmly relevant to poor prognosis following ICH (Ju and Hang 2025). END after ICH is related to a variety of factors, covering progressive brain edema, early growth expansion, brain ischemia, and hypoxia (Leira et al. 2004). Among them, neuroinflammation is a very important molecular event (Chu et al. 2023). Necroptosis is a sort of necrosis that drives innate immune responses by rupturing dead cells and releasing intracellular components (Orozco et al. 2014). Toll‐like receptor‐3 and ‐4 agonists and tumor necrosis factor (TNF) are widely existent under neurological pathological conditions (Ju and Hang 2025). These ligands can bind to TNF receptor 1 on the cell membrane and form a corresponding complex to RIPK‐1 (Micheau and Tschopp 2003). Following the phosphorylation of RIPK‐1, RIPK ‐3 is recruited, phosphorylated, and later activated (Vanden Berghe et al. 2014). RIPK‐3 acts on MLKL to phosphorylate, and the phosphorylated MLKL oligomerizes into a pore‐like structure on the cell membrane, leading to rapid cell membrane disruption and subsequently provoking an inflammatory response (Vanden Berghe et al. 2014). Overall, it is postulated that necroptosis‐associated neuroinflammation may elucidate links between MLKL, END, and poor prognosis after ICH. Thus, illuminating the mechanism underlying such intricate interplays may proffer an alternative way for decreasing END incidence and meanwhile improving clinical outcomes, as well as becoming a new research direction.

Stroke outcomes are apt to multifactorial effects and some conventional biomarkers or ratios have been extensively studied regarding their prognostic value in the neurological field (J. Liu, Luo, et al. 2024; Y. Liu, Qiu, et al. 2024; Yang et al. 2024). In a recent meta‐analysis of 12 cohort studies comprising 5042 participants, the C‐reactive protein to albumin ratio predicted mortality of patients with ischemic stroke, with AUC at 0.72 (de Liyis et al. 2024). Also, a meta‐analysis in 2025 included 77 studies, selected mortality and neurological functional status as the two outcome variables of interest, and investigated over ten clinically conventional indices, such as C‐reactive protein, D‐dimer, copeptin, S100β, white blood cell, monocyte, glucose and more, therefore finding that only S100β and copeptin had very high effect size and high certainty of evidence (Sasongko et al. 2025). In our study, serum MLKL levels had similar AUC to NIHSS scores and hematoma volume, signifying that serum MLKL may be clinically valuable in anticipation of END and poor prognosis in ICH, hopefully leading to improvement of patient outcomes or refinement of risk stratification methods. However, comparisons with other biomarkers regarding their prognostic abilities become an imperative task in the future.

There are three limitations in this study. First, although a medium sample size, which has been demonstrated to be adequate for data analyses of the current study statistically, was employed to study prognostic implications of serum MLKL in ICH, thereby finding some encouraging results that are sufficiently supportive of the conception that serum MLKL may represent a promising biomarker of prognosticating clinical outcome; the conclusions warrant to be validated in a large cohort study. Second, serum MLKL levels have been quantified at multiple time points following ICH in our study and similar predictive abilities for poor prognosis and END in terms of AUC have been revealed in those time points, while the AUCs at those time points may be greatly variable in consideration of a small sample number in such time points; So, a large cohort study is needed to confirm the conclusions. Finally, in this study, only 73 patients were allowed blood collection at several time points after ICH. Even if those 73 patients had similar baseline data to all patients, meaning that those patients may statistically represent all patients; this condition may lead to selection bias because they were not randomly selected from all patients and this mode is still different from random selection. Hence, this point is worthy of attention. Maybe, increasing the sample size is capable of promoting statistical power to some extent.

## 5 Conclusions

Serum MLKL levels are markedly increased from admission until day 15 after ICH. Serum MLKL levels are evidently correlated with NIHSS scores and bleeding amount, are independently associated with risks of END and a poor outcome at the 6‐month mark following ICH, and exhibit efficient prognostic effects in ICH. In summary, serum MLKL appears as a prognostic indicator, which may be of value to provide potential insights into the medical management of ICH.

## Author Contributions

**Yijun Ma**: conceptualization, writing–original draft, writing–review and editing, methodology, resources, supervision, visualization. **Jun Wang**: conceptualization, investigation, writing–original draft, writing–review and editing. **Chao Tang**: methodology, writing–original draft, software, data curation. **Jin Liu**: conceptualization, writing–original draft, investigation, formal analysis, data curation. **Xiaoyu Wu**: writing–original draft, validation, methodology, formal analysis, software. **Xiaoqiao Dong**: funding acquisition, writing–original draft, methodology, conceptualization, formal analysis. **Quan Du**: investigation, writing–original draft, validation, formal analysis, project administration, funding acquisition. **Wei Li**: investigation, validation, formal analysis, project administration. **Xuan Lv**: investigation, validation, writing–original draft, formal analysis, project administration. **Suijun Zhu**: writing–review and editing, writing–original draft, conceptualization, investigation, methodology, formal analysis, visualization, validation, project administration, resources, supervision.

## Conflicts of Interest

The authors declare no conflicts of interest.

### Peer Review

The peer review history for this article is available at https://publons.com/publon/10.1002/brb3.70424

## Data Availability

The datasets generated and/or analyzed during the current study are not publicly available because they are personal data, but are available from the corresponding author upon reasonable request.
